# Enhance Access to Pulmonary Rehabilitation with a Structured and Personalized Home-Based Program—*reabilitAR*: Protocol for Real-World Setting

**DOI:** 10.3390/ijerph18116132

**Published:** 2021-06-06

**Authors:** Sarah Bernard, Rui Vilarinho, Inês Pinto, Rosa Cantante, Ricardo Coxo, Rosa Fonseca, Sagrario Mayoralas-Alises, Salvador Diaz-Lobato, João Carvalho, Cátia Esteves, Cátia Caneiras

**Affiliations:** 1 Centre de Recherche, Institut Universitaire de Cardiologie et de Pneumologie de Québec, Université Laval, Québec, QC G1V 4G5, Canada; sarah.bernard@criucpq.ulaval.ca; 2 Healthcare Department, Nippon Gases Portugal, 4470-177 Maia, Portugal; ruivilarinho1@gmail.com (R.V.); inesrosendo@gmail.com (I.P.); rosa.cantante@nippongases.com (R.C.); ricardo.coxo@nippongases.com (R.C.); rosa.fonseca@nippongases.com (R.F.); prxrrjoaocarvalho@gmail.com (J.C.); catia.milene@nippongases.com (C.E.); 3 Healthcare Management Department, Hospital Quirón Salud San José, 28002 Madrid, Spain; sarimayoralas@gmail.com; 4 Nippon Gases Healthcare, 28020 Madrid, Spain; salvador.diazlobato@nippongases.com; 5 Service of Pneumology, Hospital Universitario Moncloa, 28008 Madrid, Spain; 6 Department of Pulmonology, Centro Hospitalar Universitário Lisboa Norte, Lisbon Academic Medical Center, 1649-028 Lisbon, Portugal; 7 Microbiology Research Laboratory on Environmental Health (EnviHealthMicroLab), Institute of Environmental Health (ISAMB), Faculty of Medicine, University of Lisbon, 1649-028 Lisbon, Portugal; 8 Institute for Preventive Medicine and Public Health, Faculty of Medicine, University of Lisbon, 1649-028 Lisbon, Portugal

**Keywords:** chronic respiratory diseases, COPD, exercise training, self-management, quality of life

## Abstract

Home-based models represent one of the solutions to respond to the poor accessibility of pulmonary rehabilitation (PR) services in patients with chronic respiratory disease (CRD). The main goal of this protocol is to present the implementation of the first nationwide home-based PR program—*reabilitAR*—in Portugal and the strategies to assess its benefits in patients with CRD. The program consists of 2 phases: a 12-week intensive phase and a 40-week maintenance phase (total: 52 weeks, 1 year). The intervention in both phases is composed of presential home visits and phone-call follow ups, including exercise training and the self-management educational program Living Well with COPD. Dyspnea, impact of the disease, emotional status, and level of dyspnea during activities of daily living are used as patient-reported outcomes measures. A one-minute sit-to-stand test is used as a functional outcome, and the number of steps as a measure of physical activity. To ensure safety, fall risk and the cognitive function are assessed. Data are collected at baseline, at 12 weeks, at 26 weeks and at 52 weeks. This is the first nationwide protocol on enhancing access to PR, providing appropriate responses to CRD patients’ needs through a structured and personalized home-based program in Portugal.

## 1. Introduction

The World Health Organization (WHO) indicates that hundreds of millions of people worldwide are affected by chronic respiratory diseases (CRD), including chronic obstructive pulmonary disease (COPD) [1]. These conditions associated with dyspnea, fatigue [2], together with muscle dysfunction [3], are an important cause of disability. These impairments are also associated with reduced health-related quality-of-life (HRQoL) [4,5], increased exacerbation rates [6,7,8] and mortality [9].

Pulmonary rehabilitation (PR) is an essential component in the management of CRD with the aim to improve patients’ exercise and functional capacity and psychological condition with long-term adherence to health-enhancing behaviors, including exercise training and self-management skills [10,11,12]. This behavior change also results in health economic gains due to fewer hospital admissions [13]. Thus, PR is a cost-effective treatment and has been proposed to be part of the standard care offered to patients with CRD [10]. However, despite the well-known evidence, this service is largely underutilized due to poor accessibility, with only 1% of worldwide availability of PR services for COPD patients [14]. In Portugal, this lack of access is observed due to the insufficient response to the existing programs, most of them hospital-based [15]. However, it is also observed by the barriers and limitations, which includes the absence of programs in non-urban geographic areas, lack of perceived benefits of PR, poor availability of materials and health professionals to create new programs, and lack of funding by the National Health Service (NHS) or health subsystems [15,16].

Thus, it is important to undertake actions that improve access to and delivery of PR services for suitable patients. One of the strategies in the international guidelines is the creation of new models of programs, where a home-based approach is indicated [17]. Home-based PR models, developed around the world, presented equivalent benefits to hospital-based programs in HRQoL and exercise capacity with no serious adverse events [18,19].

According to these positive results, the *reabilitAR* program was created on a value-added model of care that can contribute to increase the access of patients with CRD in Portugal. Additionally, this program provides a safe home-based PR of high quality with a direct assessment on the patients’ needs, and the environment in which PR is conducted. Its implementation makes it the first nationwide, structured, and personalized home-based PR in Portugal. The goal of this project is to present and implement the home-based PR program—*reabilitAR*—in patients with CRD. The main outcomes are to measure the short- and long-term effects of this specific home-based program on patients’ symptoms, the impact of the disease, emotional status, functional capacity, and level of physical activity. The secondary outcomes are to investigate the short- and long-term effects in the number of exacerbations and healthcare utilization. The first population included in this program were COPD patients because they represent the largest proportion of referrals in PR and much of the existing related evidence is in this population [10].

## 2. Materials and Methods

### 2.1. Design, Setting and Medical Referrals

A real-world pre- and post-intervention protocol was designed for patients with CRD. The first step in the development of the *reabilitAR* program was building an operational instruction manual according to the best practices and international guidelines [10,17,20,21]. The intervention protocol was elaborated to systematize the procedures by the *reabilitAR* team and to ensure the quality of the PR intervention, since all the procedures of the program are applied at the patients’ homes, including the assessment moments and intervention.

**Figure 1:**
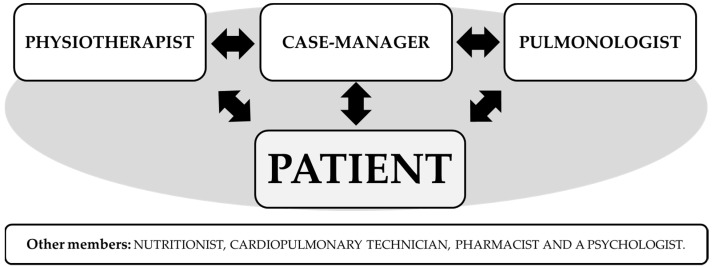
Members of the *reabilitAR* program.

The *reabilitAR* team is constituted by healthcare professionals, including a pulmonologist (responsible for the assessment of referrals to the program according to the clinical reports, for the patients’ inclusion, and the management of medical incidences and safety during the program), a respiratory nurse (the case manager, responsible for coordinating patient care by facilitating communication with the team to ensure regular progress and problem assessments, motivation and confidence building, and problem-solving support) and a trained physiotherapist (mainly responsible for the exercise training and assessments). The program also presents the collaboration of a nutritionist, a cardiopulmonary technician, a pharmacist, and a psychologist, although they do not have a direct approach in patients’ intervention, they help the other team members in the decisions making (Figure 1). The whole team works for to Nippon Gases Portugal, a home respiratory care provider which is homologated by the Portuguese NHS for the provision of domiciliary respiratory therapies (oxygen, non-invasive ventilation, and other respiratory therapies) and it is certified with scientific suitability by the National Innovation Agency (ANI) of Portugal.

Patients are referred by pulmonologists from medical consultation in hospitals and clinics, and in the future general practitioners will also be able to refer. The referral process consists of completing a document, with required information such as respiratory diagnosis, other comorbidities diagnoses, usual medication, and medical examinations (mainly, pulmonary function testing, and electrocardiogram [ECG]). During the process it is also expected that the medical doctor explains the goal and benefits of PR, according to the *reabilitAR* program. Eligible patients contact or are contacted by the *reabilitAR* team, for additional questions and to begin the integration process.

### 2.2. Eligibility Criteria

Patients are eligible if they are diagnosed with CRD. Specifically, COPD patients have to meet the following inclusion criteria: (i) diagnosis based on the Global Initiative for Chronic Obstructive Lung Disease (GOLD) criteria—postbronchodilator forced expiratory volume in 1 s (FEV_1_)/forced vital capacity (FVC) ratio <70% [20]; (ii) ECG record at rest with no significant change; (iii) written informed consent form. Exclusion criteria are: (i) presence of any clinical condition that does not allow the participation to a home-based PR program, such as, significant cardiovascular (e.g., symptomatic ischemic cardiac disease), neurological (e.g., neuromuscular dystrophy disease) or presence of musculoskeletal disease; (ii) signs of cognitive impairment (e.g., dementia) [22].

### 2.3. Data Collection

For the eligible patients, the case manager proceeds to a phone-call for a standard and structured assessment through a questionnaire, which includes socio-demographic (age, sex, educational level, marital and working status), general clinical information (medication, long-term oxygen, non-invasive ventilation, medical history, and comorbidities), eating habits and smoking habits. This data collection is sent to the physiotherapist to schedule the first home visit to complete the baseline assessment. Written informed consent is obtained prior to this baseline data collection. The outcomes measured during the first home visit are: anthropometric measures (body mass index—BMI, waist and hip circumference, waist-to-height and waist-to-hip ratio, and % body fat, % of water and fat free body mass with bioelectrical impedance measure—Tanita BC-545 N, Tanita, Amsterdam, The Netherlands) [23,24]; symptoms of dyspnea (modified Medical Research Council Questionnaire—mMRC) [25], impact of the disease (COPD Assessment Test—CAT) [26,27], emotional status (The Hospital Anxiety and Depression Scale—HADS) [28,29], level of dyspnea during activities of daily living (London Chest Activity of Daily Living—LCADL) [30], functional capacity (1-minute sit-to-stand test—1MSTS) [31], and physical activity (pedometer) [32]. To ensure the safety during the self-managed exercise training sessions, the assessment of cognitive function (Mini-Mental State Examination—MMSE) [33,34] and balance/fall risk (Berg Balance Scale—BBS and Short Form Berg Balance Scale 3-Point—SFBBS-3P) [35,36] are also included. The SFBBS-3P is applied in every patient as a screening for fall risk and only the positive ones are assessed with BBS to realize the degree of fall risk presented. This analysis is important and could change the standard scheme of visits of the *reabilitAR* program presented below, by adding visits at the beginning of the program to offer a specific exercise program for fall risk for a subsequent safe and effective self-management intervention.

During this baseline assessment is also assessed the self-efficacy for the ability to follow the exercise program regularly (≥3 days a week) over the *reabilitAR* program, considering a scale of 1 to 10, where 10 represents “very confident”. This evaluation is adapted from the LWWCOPD. Furthermore, patients are asked the expectations/objectives they pretend to achieve with the program, always reflecting the functional performance (according to the real-life situations and difficulties in usually perform their activities) [37].

After the baseline assessment, the pulmonologist of the program validates the inclusion of patients according to his analysis of the collected data (especially, ECG, comorbidities, outcomes, and fall risk), ensuring the safety for the home-based approach.

During the program, data are also collected from patients at 12 weeks (end of intensive phase), at 26 weeks (during maintenance phase) and 52 weeks (end of maintenance phase) of the program (Table 1).

### 2.4. Intervention

**Figure 2:**
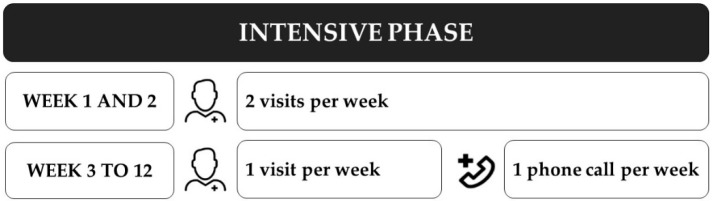
Standard number of visits and phone-calls during the intensive phase.

The program consists of 2 phases: a 12-week intensive phase and a 40-week maintenance phase for a total duration of 52 weeks (1 year). Intervention in both phases consists of a hybrid conception of presential home visits for the exercise training and the self-management sessions, and phone-call follow-ups including motivational follow-up, assessment of the clinical condition, and the progression of exercise training. The intensive phase (week 1 to 12) includes a total of 14 home visits, with more visits in the first two weeks of the program (4 visits). From the third week, one visit is replaced by a phone-call, for a total of 10 phone-calls (Figure 2). The general objective of this strategic combination of interventions is to empower the patients to reach a frequency of exercise training between 3 and 5 times per week [10], to better take control of their health behavior aiming to the self-management of their health conditions. Additionally, to provide knowledge and strategies to cope with the appearance of possible limiting factors during the program, like detecting signs and symptoms to stop exercise training.

**Figure 3:**
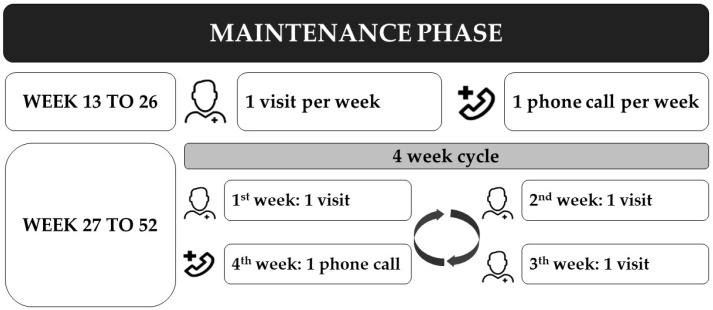
Standard number of visits and phone-calls during the maintenance phase.

In the maintenance phase, weeks 13 to 26, the intervention remains the same, with one home visit and one phone call per week. During weeks 27 to 52, starts a 4-week cycle intervention with 3 visits in the first weeks and one phone-call on the last week (Figure 3).

The *reabilitAR* program includes the self-management educational program Living Well with COPD (LWWCOPD) (available at www.livingwellwithcopd.com, accessed on 3 June 2021). Each home visit has a duration of 60 min and is delivered by a physiotherapist which includes a clinical assessment (symptoms, heart rate (HR), blood pressure (BP), oxygen saturation (SpO_2_) and perceived dyspnea and fatigue with the modified Borg scale), the teaching of the exercise training and the self-management educational intervention. The exercise training is performed according to the module “Integrating an Exercise Program into Your Life” of LWWCOPD and includes warm-up, endurance, resistance/strength, flexibility, balance training and a cool down period. Every session the physiotherapist revisits the nature of the exercise, its frequency, duration, intensity, and progression. The endurance training is performed on a portable cyclo-ergometers and steps/stairs for a target duration of 30 min per session and the training intensity measured and limited to a Borg dyspnea or fatigue score of 4 to 6 [10,18]. The resistance/strength training is performed using a variety of tools such as dumbbells, ankle weights and elastic bands for up to 20 min with exercises of the major upper limbs, lower limbs, and trunk muscle groups. Initial loads equivalent to one that evokes fatigue after 10–12 repetitions, with 1 to 3 sets [10]. The flexibility and balance training component are performed according to the patients’ need. Flexibility training is done using stretching exercises of the major muscle groups [10] and balance training with postural exercises, transition, and gait exercises [38,39]. During the program, progression of the training intensity is also tailored according to the perceived dyspnea and fatigue using the modified Borg scale. All the material mentioned above for the exercise training is provided for each patient by the homecare provider responsible for the program. Additionally, a modified Borg scale and a diary is offered to register their exercise training during the unsupervised sessions. It is also asked to register any symptoms worsening or exacerbations, changes in medication or other aspects that they care to report to the physiotherapist or the case manager, according to the self-management concept.

The self-management educational intervention is based on the modules of LWWCOPD and is performed especially during the intensive phase of the program. Beside the module on exercise training, each patient receives one other module entitled “Being Healthy with COPD”, that includes every educational topic concerning self-management (4). If needed, the other modules presented in LWWCOPD are available according to the patients’ specific needs. A detailed description and the selected order of the themes are presented in Table 2.

Patients also receive a pedometer and recommendations on how to use it to increase physical activity.

**Figure 4:**
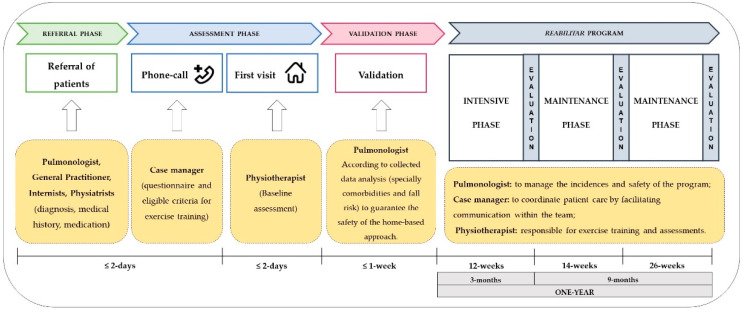
Overview of the timeline and procedures of the *reabilitAR* program.

An overview of the timeline and procedures of the *reabilitAR* program is available in Figure 4.

### 2.5. Statistical Analysis

After the assessment moments (12 weeks, 26 weeks, and 52 weeks), a report with the total scores of patient-reported outcomes measures (mMRC, CAT, HADS, LCADL), the performance of the functional test outcome (1MSTS), physical activity (number of steps), and the number of exacerbations and healthcare utilization are registered along with the baseline data for a term of comparison. According to the available evidence, established minimal clinically important differences (MCID) are also included according to the respective diagnosis of CRD. This report is sent to the referrer doctor on each assessment moment.

In order to detect the benefits of the *reabilitAR* program in patients with COPD, data from the study of Vaidya and colleagues (2016) [31], by using the differences in functional capacity assessed with the 1MSTS in patients undergoing PR, was used to calculate the effect size and to estimate the sample size. The next formula was used:

Effect size = [(mean post − mean pre)/SD difference](1)

where “mean post” is the mean of the number of repetitions after PR, “mean pre” is the mean of the number of repetitions before PR, and “SD difference” is the standard deviation of the mean change. The difference between post- and pre- number of repetitions achieved in Vaidya study (2016) was 3.8 repetitions and the SD of this difference was 4.2 [31], resulting in an effect size of 0.90. Therefore, a total sample size of 14 patients will be considered to explore the first results of the *reabilitAR* program. For this purpose, we will include 25 patients in our recruitment since PR programs have considerable dropout rates, varying between 20 and 40% [40]. We are predicting to reach this recruitment goal between 6 months and 1 year.

Statistical analyses will be performed using IBM SPSS Statistics (IBM Corporation, North Castle, NY, USA) with a level of significance of 0.05. Descriptive statistics (frequencies, means and standard deviations, medians, and interquartile ranges) will be used to describe the patients. The benefits of the *reabilitAR* program will be verified using paired *t*-test or Wilcoxon signed-rank tests, accordingly to data normality. Differences between the different time points will be assessed using a one-way analysis of variance with repeated measures to establish the significant effects of time. With a significant effect of time, post hoc analyses will be made with pairwise comparisons using the Bonferroni correction.

## 3. Discussion

This real-world protocol presents the first nationwide home-based PR program in Portugal, focusing on enhancing patients’ access to PR. During the development of this program, the collaboration of national and international clinical experts in PR has ensured the highest standards, with a structured and personalized intervention. These strategies allowed to increase the quality of *reabilitAR* program content, which is the most important factor in the development of PR programs. This statement is proved by the evidence, where the benefits of the structured home-based models are already comparable to those obtained with the hospital-based PR [18], which makes the content of a PR program more important in determining efficacy than the setting. Additionally, to ensure these highest standards, the well-known, validated, and effective self-management program LWWCOPD [18,41], focusing on behavior change, has been integrated directly in our program.

One of the important steps in the development of our program was the choice of the outcomes measures to demonstrate its benefits, since this program is totally conducted in the patients’ homes, including the assessments moments and intervention. The mMRC, CAT and LCADL are recommended by the PR guidelines [10] and their administration is simple, clear, and not time-consuming. The HADS facilitates the identification of barriers influencing the exercise self-efficacy and factors that can help create important strategies for the self-management home intervention (e.g., identifying stressors to break the anxiety-breathlessness cycle). In addition, this scale provides important information about anxiety and depression symptoms, associated with mental illness, which can reduce HRQoL, increase the risk of exacerbations and mortality [42]. The mental health illness topic is receiving the more attention and priority in the evidence on interventions, including PR, in patients living with COPD [43].

For the assessment of the physical component, the 1MSTS was chosen because it requires limited equipment and space [44], unlike the most used tests in PR for exercise and functional capacities (e.g., the field walking tests) [45]. Additionally, this test reflects a common activity in daily living and has good measurement properties to PR [31,37,46,47,48,49,50]. Additionally, the presence of an ECG record at rest, with no significant changes, was also considered an important and mandatory eligible criterion to ensure safety, since the cardiovascular disease in CRD, especially in COPD, is one of the most prevalent comorbidities [51]. The assessment of physical activity by the record of the number of steps is also an important outcome, since people with CRD, especially in COPD, present low levels of physical activity [52], which are associated with a higher risk of comorbidities [53].

Another important aspect that the *reabilitAR* program could offer is the full assessment of the health experience of patients with CRD, according to the International Classification of Functioning, Disability, and Health (ICF) [37]. Traditionally, the assessment of the patients in the hospital-based programs is only based on two domains (body functions and structures, and activities) with laboratory-based tests and exercise test outcomes, that need to be performed in a standardized environment. However, their assessments are not always representative of patients’ functional capacity and true ability to fulfill their social roles in the real-life situations [37,54]. The home-based approach is, in fact, an important opportunity to add a direct observation and intervention of daily life activities in patients’ real environment supported by functional measurements.

In the literature, we can find evidence that indicates that home-based programs with longer durations can have longer-lasting benefits [18]. Additionally, the longitudinal design (1-year) of the *reabilitAR* program complies one of the recommendations of the ATS/ERS Policy Statement for the long-term adherence to health enhancing behaviors, as a key goal of PR for patients with CRD [10]. Currently, the reality of the PR in Portugal is mostly on hospital-based programs in centralized urban areas [15], where only the assessment of their short-term effects is performed, without monitoring the maintenance of the results over time. To overcome this barrier, one of the important aims of our program is to provide an integrative and patient-centered care with a possible care transition for the ongoing rehabilitation process after hospital-based PR.

This initiative to develop an alternative model of PR reflects the Portuguese Government’s concerns to provide appropriate responses to CRD patients’ needs and increase their access to PR according to their geographic distribution [55]. It is also representing a comprehensive and well-resourced home-based program model that makes evidence-based PR more accessible and acceptable to patients and payers [17]. Furthermore, this program aims to offer a nationwide service, to improve the patients’ HRQoL by reducing the disabilities associated with the disease, and to improve patients’ self-efficacy and self-management of their health condition with the appropriate use of different health resources.

It is important to acknowledge the limitations of this protocol. With the intent to implement a real-world home-based PR, patients’ referral to the program is mandatorily done through a medical doctor, rather than directly through the program. Therefore, the number of patients validated to the program is exclusively dependent on the number of medical referrals. In order to overcome this barrier, this program also has the objective to raise awareness amongst health professionals about PR, its benefits, the target population, referrals, and the available program. Another concrete limitation is the option for exercise training. According to the Portuguese orientation of the Directorate-General of Health for PR [56], grounded by ATS/ERS guidelines [10], the framework recommended for the intensity of the endurance training (60% of maximal work rate on the cardiopulmonary exercise testing or 80% of the average speed on 6MWT) is not possible to implement in context of the *reabilitAR* program. Nevertheless, the exercise intensity is selected by the perceived dyspnea and fatigue on the modified Borg scale [10,18], which is widely used tool for self-monitoring and self-regulating exercise for patients during the unsupervised sessions and recommended in the LWWCOPD program.

The implementation of the *reabilitAR* program represents the ideal opportunity to explore future research to advance evidence-based in home-based PR. One proposal is to assess the maximal voluntary muscles contraction with handheld dynamometer to explore the characterization of the muscular strength and its short- and long-term effects with home-based PR in patients with CRD. The assessment of this outcome is important since it is served as a predictor of mortality [3]. Another proposal is to assess the long-term effects in behavior change of physical activity with the gold standard measure using an accelerometer.

## 4. Conclusions

We expect that the *reabilitAR* program will enhance the patients’ access to PR in Portugal and provide an evidence-based insight into the positive impact on symptoms, emotional status, functional capacity, and physical activity in patients with CRD.

## Figures and Tables

**Table 1:** Outcomes assessed in each time point of the *reabilitAR* program.

Timepoint	Baseline T0	Post 12 Weeks T1	Post 26 Weeks T2	Post 52 Weeks T3
Intervention	Home-Based Pulmonary Rehabilitation—*reabilitAR*			
Outcomes				
Anthropometric measures	X	X	X	X
Impact of the disease	X	X	X	X
Emotional status	X	X	X	X
Balance and fall risk	X	X	X	X
Cognitive function	X			
Functional capacity	X	X	X	X
Number of exacerbations and hospital admissions	X	X	X	X
Level of physical activity	X	X	X	X

**Table 2:** Modules and educational topics proposed for the home visits of the intensive phase (14 visits).

Self-Management Educational Intervention		
Visit	Module	Themes
1	Integrating an Exercise Program into Your Life	Exercise program, pursed-lip breathing technique and scale of perceived exertion
2	Being Healthy with COPD—Preventing your Symptoms and Taking Your Medication	Anatomy, physiology and cause of COPD and factors that can make your symptoms worse
3		Medications and inhalation techniques
4	Managing your Breathing and Saving Your Energy	Understanding how breathing works, reducing shortness of breath (breathing techniques and body positions)
5		Clearing your airways (coughing tecniques, active cycle of breathing technique and devices)
6		Clearing your airways (devices)
7		Applying energy conservation principles
8	Review of previous themes (doubts) and reinforce the importance of medications and breathing techniques/body positions for shortness of breath	
9	Managing Your Stress & Anxiety	Identifying the stressors in your life and understanding your reaction, breaking the anxiety-breathlessness cycle
10	Associate the topics of visit 4 and visit 9	
11	Managing Your Stress & Anxiety	Integrating relaxation exercises and applying rules to live a healthier life
12	Integrating a Healthy Diet into Your Life	The link between COPD and what you eat, maintaining a healthy weight and healthy and balanced eating
13	Keeping a Healthy and Fulfilling Lifestyle	Being healthy, quitting smoking (optional), sleeping better, satisfying sex life, leisure activities and travelling
14	Review of previous themes (doubts)	

## Data Availability

None.
